# Targeting SARS-CoV-2 Mpro and PLpro by Repurposing Clinically Approved Drugs

**DOI:** 10.3390/v17121564

**Published:** 2025-11-29

**Authors:** Qiaoyu Fang, Meng Lu, Derong Chen, Liangxu Xie, Wenxu Hong, Zhang Zhang, Xuqiao Hu

**Affiliations:** 1 Shenzhen Center for Chronic Disease Control and Prevention, Shenzhen Institute of Dermatology, Shenzhen 518020, China; fangqy999@sina.com (Q.F.); menglu11161003@163.com (M.L.); nemochen229@163.com (D.C.); szbloodcenter@hotmail.com (W.H.); 2 Institute of Bioinformatics and Medical Engineering, School of Electrical and Information Engineering, Jiangsu University of Technology, Changzhou 213000, China; xieliangxu@jsut.edu.cn

**Keywords:** SARS-CoV-2, Mpro, PLpro, oxytocin, cephalosporin drugs

## Abstract

SARS-CoV-2 virus contains two highly conserved domains, the papain-like protease (PLpro) and main protease (Mpro), which play important roles in virus replication, immune suppression, and the induction of inflammation in host tissue. In this study, we applied small-molecule chip screening, enzymatic assays, SARS-CoV-2 spike pseudotyped virus detection and molecular docking to find potential Mpro or PLpro inhibitors. Two small molecules, oxytocin and risedronate sodium, stood out in drug repurposing. Oxytocin and risedronate sodium were shown to influence the activities of Mpro and PLpro, thereby preventing the virus from replication, which may alleviate SARS-CoV-2 infection. Thus, oxytocin, risedronate sodium, and cephalosporins may expand the drug library for treating coronavirus infection.

## 1. Introduction

Despite the fact that the COVID-19 pandemic has been ongoing for more than five years and that vaccines against the SARS-CoV-2 virus have been created, the appearance of SARS-CoV-2 variants and drug-resistant genetic variants continues to highlight the urgent need for antiviral drugs, posing a concern for global health security.

SARS-CoV-2 virus has multiple open reading frames for the production of structural and functional proteins [1]. ORF1a and ORF1b are two polyproteins produced by the two coding sequences of the open reading frame 1. Nonstructural protein 5 (NSP5) encodes the main protease (Mpro) and Nonstructural protein 3 (NSP3) encodes the papain-like protease (PLpro), which act as protein scissors and cleave ORF1a and ORF1b into 16 functional proteins. Hence, Orf1a and Orf1b encode 16 nonstructural proteins that are involved in the transcription and replication of the virus [2,3].

Mpro is a key enzyme of coronaviruses and plays a pivotal role in mediating viral replication and transcription. It consists of three domains, in which domains I and II are β-barrels, while domain III mainly consists of α-helices. Cys145 and His41 are the catalytic sites of Mpro, offering a breakthrough for the development of drugs targeting SARS-CoV-2 [4]. Other prominent amino acid residues at this site include Gly143, Ser144, Thr26, Thr24, and Leu27 [5]. Except for Mpro, a highly conserved domain called PLpro is another potential therapeutic target of SARS-CoV-2 [6]. By cleaving the nsps at several junctions, PLpro plays a crucial part in viral replication [7]. The active sites of PLpro contain a ubiquitin-binding site (MET208/PRO247 and VAL70Ub) and a known BL2 groove pocket (PRO248, TYR264, and TYR268) [8].

Based on the structure and active sites of Mpro and PLpro, several inhibitors have been discovered and clinically used. For example, the Mpro inhibitor nirmatrelvir has already received approval for treating COVID-19. However, the potential for developing drug resistance [9] necessitates the need for next-generation therapies and an expanded drug database. A promising study demonstrated the superior therapeutic efficacy of ML2006a4, which was not sensitive to any of the several naturally occurring mutations [10]. Additionally, an α-ketoamide-based peptidomimetic inhibitor of Mpro, RAY1216, was allowed to treat COVID-19 in China, and RAY1216 is postulated to covalently attach to the catalytic Cys145, providing exceptional antiviral activity [11]. Rao et al. (2005) described Mpro as a highly flexible structure with S1 pocket [4]. Subsequently, the researchers identified an inhibitor (N3) and determined the protein-molecule complex [3]. In addition, the β-lactams comprising penicillin esters were found to be potential inhibitors of Mpro [12].

To identify clinically approved medications that may have an inhibitory effect on Mpro and PLpro, a microarray containing 3145 small molecules was employed in conjunction with molecular docking analysis. Both Mpro and PLpro are highly conserved domains, and a few of the approved drugs were shown to have an inhibitory effect on these enzymes, a result that may expand the library of medicines available for COVID-19 treatment. The structure of potential candidates can be modified to create new antivirals for the screening of SARS-CoV-2 variants and drug-resistant mutants.

## 2. Materials and Methods

### 2.1. Preparation of Labeled Proteins

Briefly, SDS-PAGE was used to assess the purity of target proteins (Novo Protein, Shanghai, China), and the Bradford assay [13] was used to quantify the concentration. Proteins were labeled with Atto 647N dye (Sigma-Aldrich, St. Louis, MO, USA), and the dot plot was used to verify the labeling efficiency. First, prepare the protein stock solution by mixing 100 µL of 1 M sodium bicarbonate solution (pH approximately 8.5 to 9.0) with 900 µL of the target protein solution to obtain 1 mL of protein labeling stock solution, with a protein concentration of 2–10 mg/mL. Second, prepare the dye stock solution by adding anhydrous DMSO to the ATTO 647N NHS ester vial to form a 10 mM stock solution. Vortex to mix thoroughly. Third, perform the conjugation reaction by mixing Solution B (dye) and Solution A (protein) at a 10:1 molar ratio. Step 4: Purify the dye-protein conjugate using a Sephadex G-25 column (Sigma-Aldrich, St. Louis, MO, USA). Finally, measure the OD (optical density) at 280 nm and the dye’s maximum absorption wavelength (for ATTO 647N NHS ester, ƛmax = 645 nm) to determine the absorption rate [14].

### 2.2. Small Molecule Microarray

Small-molecule microarrays were purchased from Bochong Biological Technology (Bochong, Guangzhou, China). The microarray contained 3145 drugs, including FDA-approved medications, Chinese herbal medicine, and small-molecule inhibitors. The experimental incubation process was performed as previously described [15,16,17,18]. Briefly, the microarray was removed from the −80 °C freezer and incubated with blocking solution at room temperature on a shaker for 1 h. The chip was cleaned once with 0.5× cleaning solution and ultrapure water. After drying, the chip was detected at 635 nm to collect the background signal. The chip was subsequently cleaned once with the cleaning solution and gently incubated with 5 μg/mL labeled protein solution for 1 h on a rotator. This was followed by three washes with the cleaning solution and two washes with ultrapure water. The chip was subsequently centrifuged and detected at 635 nm. All of these processes were completed in the dark at room temperature.

### 2.3. Microarray Data Processing

GenePix Pro v6.0 (Molecular Devices, Sunnyvale, CA, USA) was used to extract the fluorescent intensity from the microarray images. The F635 median and B635 median (the median of the foreground and the background signals) were used to define the signal-to-noise ratio (SNR), which was calculated as the average of three replicates. Based on the mean and SD of SNR, the cutoff threshold was set to screen potential positive small molecules, which was calculated using the formula cutoff = mean + 1.96 × SD (95%CI). The SNRs of the potential candidate molecules were higher than the threshold.

### 2.4. SARS-CoV-2 PLpro and Mpro Enzymatic Assays

To determine the inhibitory effect of 13 prioritized small molecules, a FRET-based enzymatic assay (Beyotime, Shanghai, China) was applied for Mpro as previously represented [19]. Briefly, 2 μL of the fluorogenic peptide substrate was added to each well. The reaction mix was incubated for five minutes at 37 °C in the dark after the substrate was added as the last ingredient. A Thermo Varioskan LUX (Thermo Fisher Scientific, Waltham, MA, USA) spectrophotometer was then used to detect the fluorescence of each well (325/393 nm). For each chemical, the percentage of inhibition was determined using the formula: Inhibition% = (RFU_100%Enzyme_ − RFU_sample_)/(RFU_100%Enzyme_ − RFU_blank_) × 100%. Each experiment was carried out twice, and GraphPad Prism (https://www.graphpad.com, accessed on 19 June 2025) was used to analyze the experimental data.

The SARS-CoV-2 PLpro Inhibitor Screening Kit was provided by tiangsa (tjs bio, Beijing, China). The FRET fluorescently labeled peptides containing the LXGG sequence were used as substrates. The fluorescence at 460 nm could be generated after the peptides were cleaved by the enzyme when excited at 360 nm. The strength of the inhibitors was determined by detecting the changes in fluorescence values. Prepare 4× PLpro enzyme solution, 2× PLpro reaction buffer, ultrapure water, solvent, and inhibitor controls. Add the reagents according to the groups, and finally add 20 μL of PLpro fluorescent substrate solution to each well. Incubate at 25 °C for 30 min with gentle shaking. After the incubation, fluorescence was detected (340/460 nm). Finally, calculate the inhibition rate: inhibition rate = [1 − (fluorescence value of inhibitor well − fluorescence value of blank well)/(fluorescence value of well without inhibitor − fluorescence value of blank well)] × 100%. The inhibition rate was conducted with drug dosage of 10 μM.

### 2.5. Protein Preparation for Molecular Docking

The three-dimensional models were downloaded from the RCSB Protein Data Bank (PDB), while the PDB IDs are 8DZB for SARS-CoV-2 Mpro and 7TZJ for PLpro (http://www.rcsb.org/, accessed on 8 December 2024) [20]. Autodock4 and AutoDockTools (Version 1.5.6) software was used to process the proteins, which included adding hydrogen atoms and removing water molecules. Protein files in pdbqt file format were prepared for molecular docking.

### 2.6. Ligand Preparation for Molecular Docking

The structures of the ligands were obtained from PubChem [21]. AutoDock software was used for ligand processing, including hydrogenation, water removal treatment, root detection, and torsion calculation, and the ligand files were prepared in pdbqt file format.

### 2.7. Molecular Docking

The docking of SARS-CoV-2 Mpro and PLpro with potential ligand candidates and the analysis of binding affinity and protein-ligand interactions were performed using AutoDock Tools [22]. The grid box center of the proteins was designed, and the same mesh box size was used for all docking runs to obtain different docking conformations. PyMOL (version 3.7.2) and PLIP (https://projects.biotec.tu-dresden.de/plip-web/plip/index, accessed on 28 August 2024) were used for visualization and docking site identification.

### 2.8. Cytotoxicity Assay

The cytotoxicity assay was conducted as previously reported [23]. ACE2-HEK293T cells were seeded at 5 × 10^4^ cells per well in 96-well plates and cultured overnight. Subsequently, a final concentration of 300 μM, 100 μM, 30 μM, 10 μM, and 3 μM of cefaclor, cefotetan, oxytocin, and risedronate sodium was added to the wells, followed by another 24 h of incubation. Cell viability was then assessed using the Cell Counting Kit solution (KeyGEN BioTECH, Nanjing, China), and the absorbance at 450 nm was measured with a Varioskan LUX plate reader (Thermo Fisher Scientific, Waltham, MA, USA). The cell survival rate was calculated from absorbance values.

### 2.9. SARS-CoV-2 Spike Pseudotyped Virus Detection

The SARS-CoV-2 spike pseudotyped virus assay was performed as previously described [24]. Briefly, ACE2-HEK293T cells were seeded into white 96-well plates at a density of 5 × 10^4^ cells per well in 50 μL of culture medium and incubated for 2 h at 37 °C under 5% CO_2_. After incubation, 25 μL of supernatant was aspirated and replaced with 25 μL of fresh medium containing the indicated drug concentrations. Following a further 2-h incubation, the cells were inoculated with 5 μL of SARS-CoV-2 spike pseudotyped virus (Sino Biological Inc., Beijing, China) for 4 h. Subsequent supplementation with 100 μL of culture medium per well was followed by 6–8 h of incubation, after which the medium was refreshed with 200 μL per well of freshly prepared medium. Cells were cultured for an additional 48 h, after which the medium was removed and lysed using 100 μL per well of luciferase assay buffer (Beyotime, Shanghai, China). Luciferase activity was measured at an emission wavelength of 560 nm upon addition of the luminescent substrate, using a microplate reader.

## 3. Results

### 3.1. Large-Scale Screening of Active Compounds Based on Small-Molecule Microarray

**Figure 1:**
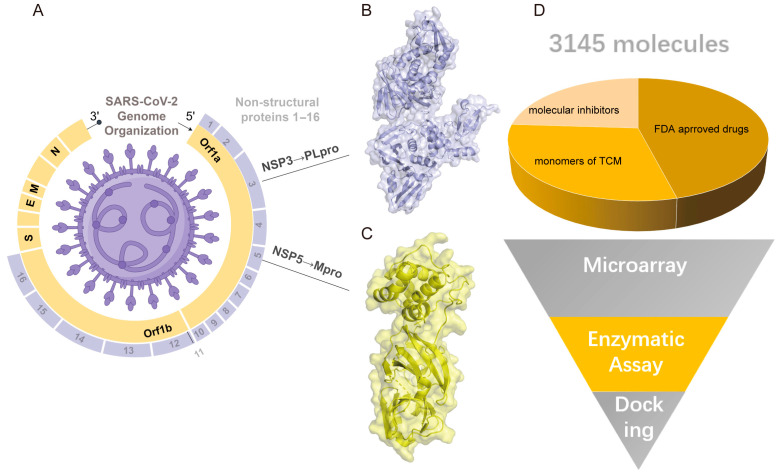
The illustration of target proteins and the screening of small molecules. (**A**) A SARS-CoV-2 genomic structure. (**B**) The SARS-CoV-2 PLpro crystal structure (PDB:7TZJ). (**C**) The SARS-CoV-2 Mpro crystal structure (PDB:8DZB). (**D**) The schematic diagram of screening the targets of SARS-CoV-2 Mpro and PLpro.

In this study, a large-scale screening method for drugs targeting Mpro and PLpro was developed based on a high-throughput microarray. As shown in Figure 1A–C, Mpro and PLpro are encoded by NSP3 and NSP5 in Orf1a, and the recombinant 2019-nCoV Mpro and PLpro were obtained from a de novo protein. The concentrations of Mpro and PLpro before labeling, as determined by Bradford assay, were 916 μg/mL and 3.4 mg/mL, respectively. After labeling, their concentrations were determined using grey scale index. The purity of the two proteases was 97% and 92.3%, respectively (Figure S1A,D). Gradient-diluted Atto 647N-labeled proteins were used to examine sensitivity and specificity, and the minimum detection limits were 0.63 ng (Figure S1B,E). Moreover, the correlation coefficients (R2) of triplicate spots for Mpro were 0.96, 0.96, and 0.93, while they were 0.96, 0.97, and 0.96 for PLpro (Figure S1C,F).

### 3.2. The Small Molecule Microarray Analysis for Drug Repurposing

Based on the experiments described above, both the protein samples met the requirements for the microarray experiment. The small-molecule microarray contained 3145 small molecules, of which 1000 were Chinese medicinal compounds. In addition, 1500 molecules were FDA-approved drugs, and approximately 800 were small-molecule inhibitors (Figure 1D). Through microarray screening, 24 drug candidates were shortlisted for further analysis, which was performed using molecular docking. In the final step, based on the drug-protein complex interaction, the most promising small molecules, oxytocin, risedronate sodium, trifluridine, and sodium salt of valproic acid, were focused on in the following research.

**Figure 2:**
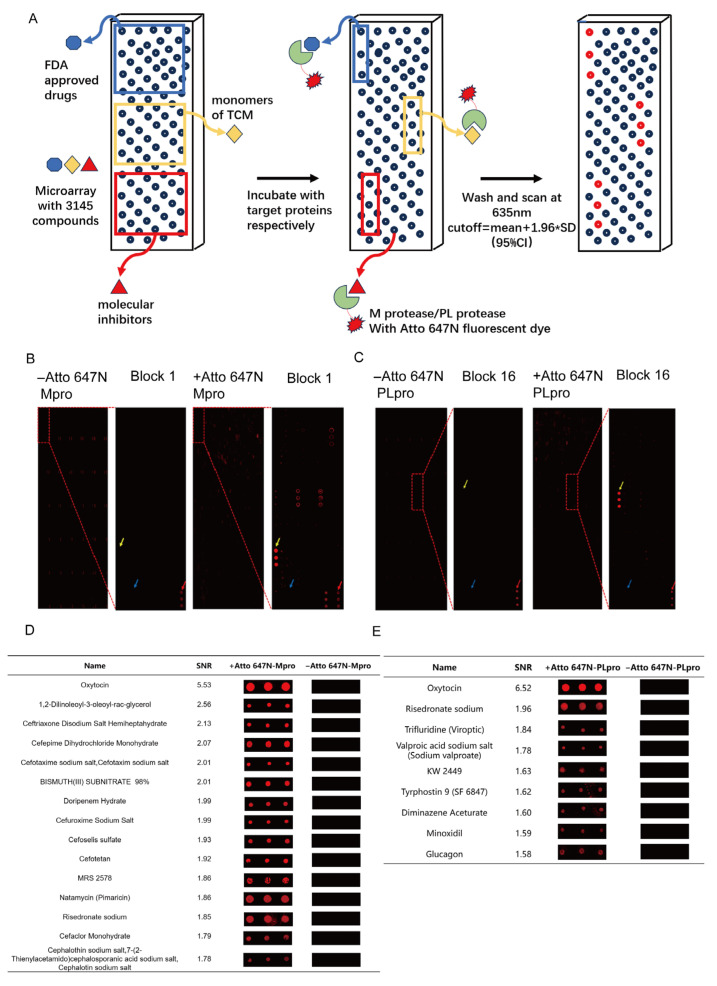
Screening of potential ligands of target proteins through micro-molecule chips. (**A**) The fluorescent detection procedure of labeled proteins that interact with small-molecule microarray. (**B**) The fluorescence images of the microarray before (**left**) and after (**right**) Atto 647N-labeled Mpro incubation. (**C**) The fluorescence images of the microarray before (**left**) and after (**right**) Atto 647N-labeled PLpro incubation. In (**B**,**C**), the red arrow demonstrates a positive control (650 fluorescent dye), the blue one shows a negative control (DMSO), and the yellow one presents a positive small molecule. (**D**) The top 15 small molecules that bind with Mpro were selected according to the SNR index. (**E**) 9 small molecules bind with Mpro according to the SNR index. The signals of Atto 647N were scanned at 650 nm, + with Atto 647N labeled-protein; −, with 650 nm fluorescent dyes.

Figure 2A depicts the workflow of the interaction between the protein samples and small-molecule microarrays. The fluorescence of the proteins remaining on the chip provided information on the interactions between the small molecules and proteins. Figure 2B,C show randomly selected fluorescent images of the microarray before and after incubation with Mpro or PLpro, respectively. The positive fluorescent dots are indicated by yellow arrows. The fluorescent-positive small molecules (39 for Mpro and 9 for PLpro) are shown in Table S1. The top 15 and top 9 drug candidates shortlisted for Mpro and PLpro with the highest fluorescent SNR are listed in Figure 2D,E.

### 3.3. The Binding Energy Analysis of Active Compounds from Chip Screening

**Figure 3:**
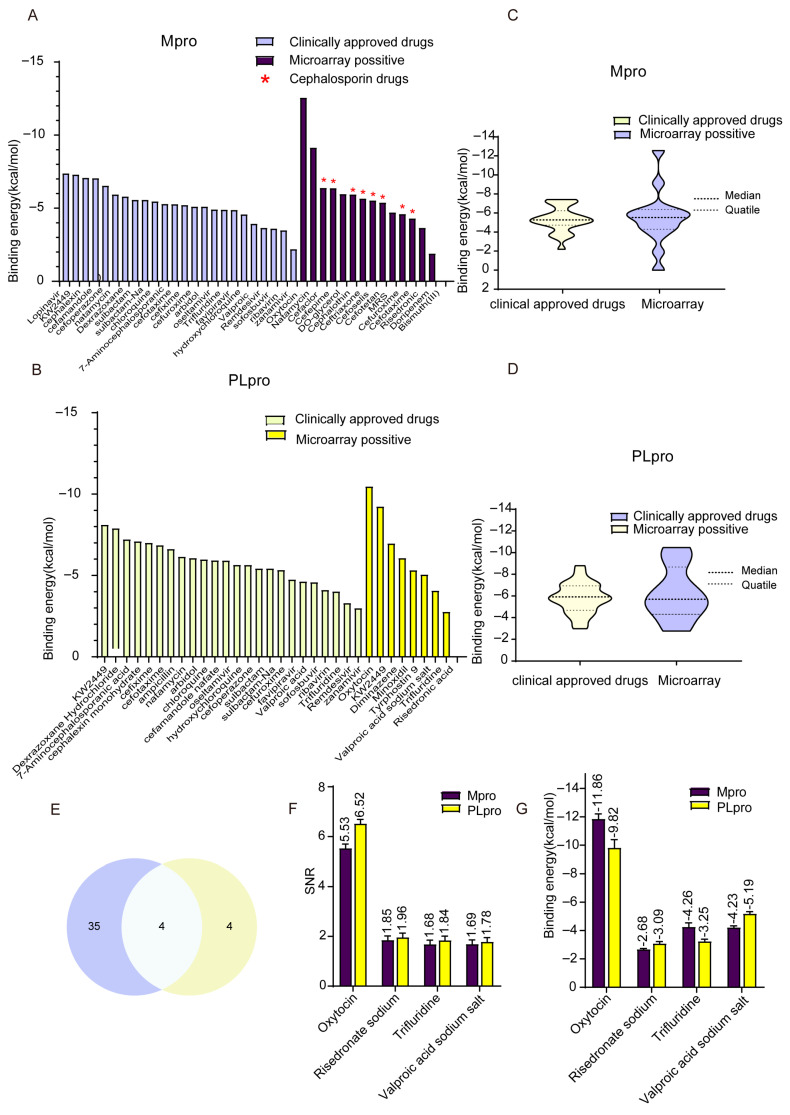
The screening of potential ligands of Mpro and PLpro with molecular docking. Based on the chip screening, the binding affinities (kcal/mol) of the top 15 candidates for Mpro and the top 8 candidates for PLpro. (**A**,**B**) The bar chart of binding affinities (kcal/mol) for FDA-approved antiviral drugs (bright color) and chip-screened drugs (deep color) for Mpro and PLpro. (**C**,**D**) The violin plot of binding affinity range (kcal/mol) of clinically approved drugs and microarray-screened drugs for Mpro and PLpro. (**E**) The Venn plot of the common potential ligands of Mpro and PLpro. (**F**,**G**) The SNR index and the binding energy (kcal/mol) of the common potential ligands of Mpro and PLpro. Each data point represents the mean of triplicate assays with ±SEM.

Molecular docking analysis was applied to estimate the possible affinity of the molecules scanned by microarray and target proteins. As listed in Table S2, the crystal structures of Mpro (PDB:8DZB) and PLpro (PDB:7TZJ) were used for docking. Fourteen out of fifteen molecules for Mpro and eight out of nine molecules for PLpro were shown to bind with the respective proteases, with scores ranging from −11.86 to 0 kcal/mol (Figure 3A,B). The docking of oxytocin with Mpro and PLpro showed the lowest binding scores at −11.86 kcal/mol and −10.47 kcal/mol, respectively. Moreover, strong binding affinities were indicated by binding scores of less than −7 kcal/mol for KW-2449 with PLpro and natamycin with Mpro. The bar chart (Figure 3A,B) and violin plot (Figure 3C,D) show a comparison of the binding scores of microarray-screened drugs with the FDA and Pharmacopeia clinically approved drugs. As shown in Figure 3A, the cephalosporins showed binding affinities with Mpro. In a comparison between ligands with Mpro and PLpro, the median did not show significant fluctuations, with a wide gap between the highest and lowest docking scores in the microarray group. In the score comparison of PLpro, the interquartile range in the microarray group was wider than that of the clinically approved drug group. A Venn diagram was created with 39 potential ligands of Mpro and 8 potential ligands of PLpro in order to pinpoint the common molecules (Figure 3E). The SNR (Figure 3F) and docking scores (Figure 3G) of common molecules—oxytocin, risedronate sodium, trifluridine, and sodium salt of valproic acid—are displayed. Oxytocin exhibited the highest binding affinity towards Mpro (SNR: 5.53, docking score: −11.86) and PLpro (SNR: 6.52, docking score: −9.82).

### 3.4. Small Molecules with Inhibitory Ability Against Mpro/PLpro Enzymes

**Figure 4:**
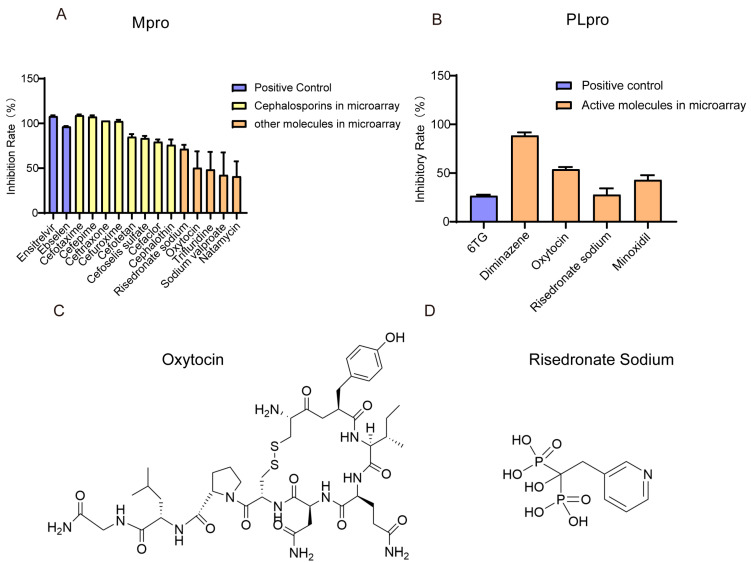
The inhibitory effect of microarray positive small molecules on Mpro and PLpro, and the structures of oxytocin and risedronate sodium. (**A**) Bar chart of the inhibition rate of small molecules on Mpro. (**B**) Bar chart of the inhibition rate of small molecules on PLpro. Each data point represents the mean of triplicate assays with ±SEM. (**C**) The chemical structures of oxytocin. (**D**) The chemical structures of risedronate sodium.

**Figure 5:**
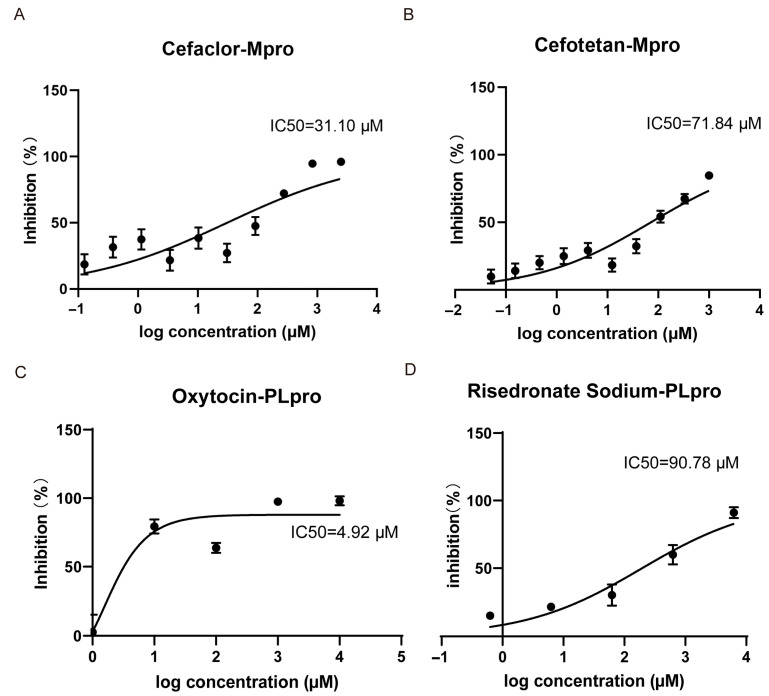
The dose–response curve of active compounds on Mpro and PLpro. (**A**,**B**) The dose–response curve of cefaclor and cefotetan on Mpro. (**C**,**D**) The dose–response curve of oxytocin and risedronic acid sodium on PLpro. Each data point represents the mean of triplicate assays with ±SEM.

In vitro enzymatic assays demonstrated that cephalosporins are potent inhibitors of Mpro. In contrast, oxytocin and risedronate sodium exhibited a broader inhibition profile, showing activity against both Mpro and PLpro. As shown in Figure 4A, both the clinically approved Mpro inhibitor ensitrelvir and the laboratory-proven molecule ebselen inhibited Mpro with inhibition rates of 108% and 96.5%, respectively. Cephalosporins, including cefotaxime, cefepime, ceftriaxone, and cefuroxime, showed an inhibitory effect comparable to that of the positive drugs, and the inhibitory rates of cefotetan, cefoselis, cefaclor, and cephalothin were 85%, 83.5%,79.5%, and 76%. The inhibitory rates of risedronate sodium, oxytocin, trifluridine, sodium salt of valproic acid, and natamycin were 71.5%, 50.4%, 48.51%, 42.43%, and 40.98%, respectively. The concentration of molecules detected in Figure 4A were 5 mM for ensitrelvir, 0.5 mM for ebselen, 9.5 mM for cefotaxime, 13 mM for cefepime, 10 mM for cefuroxime, 1 mM for cefotetan, 2.5 mM for ceftriaxone, cefoselis, cefaclor, oxytocin, 7.5 mM for cephalothin, 1.25 mM for risedronic acid sodium, 15 mM for trifluridine and sodium salt of valproic acid, and 0.5 mM for Natamycin. In addition, Figure 5A,B show that the IC_50_ of cefaclor and cefotetan for Mpro were 31.10 μM and 71.84 μM.

As for PLpro (Figure 4B), the competitive inhibitor 6-TG exhibited a moderate inhibitory rate of 26.67% at 100 μM, while diminazene showed a significant inhibitory effect with a rate of 88.59%. Oxytocin, risedronate sodium, and minoxidil inhibited PLpro with inhibition rates of 53.93%, 27.85%, and 42.95%. The concentration of molecules detected in Figure 4B activity bar chart was 10 μM. To be noticed, oxytocin and risedronate sodium demonstrated inhibitory effects against both Mpro and PLpro (Figure 4C,D). Furthermore, as illustrated in Figure 5C,D, the IC_50_ of oxytocin and risedronate sodium against PLpro were 4.92 μM and 90.78 μM.

### 3.5. The Inhibition Effect of Molecules on SARS-CoV-2 Spike Pseudotyped Virus

**Figure 6:**
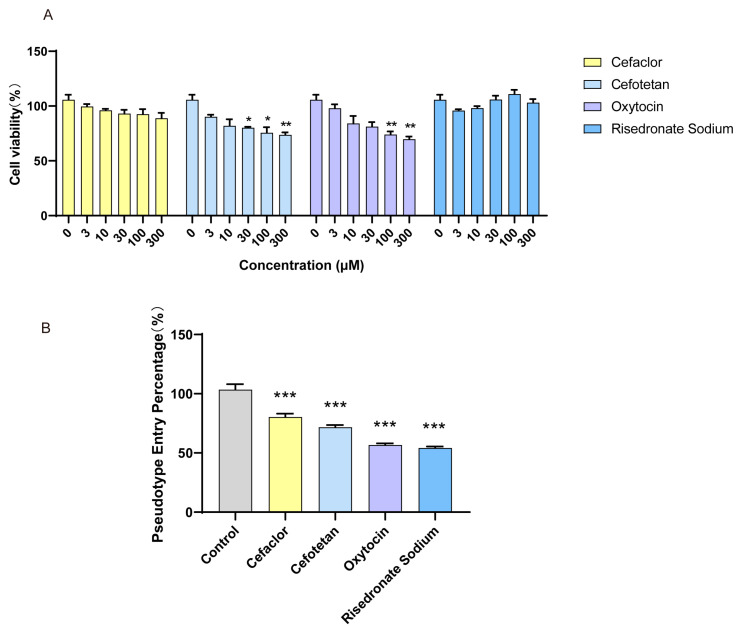
SARS-CoV-2 spike pseudotyped viral entry into ACE2-HEK293T cells is inhibited by microarray-positive small compounds. (**A**) The cytotoxicity assay of cefaclor, cefotetan, oxytocin, and risedronate sodium on ACE2-HEK293T. (**B**) Bar graph of the SARS-CoV-2 spike pseudotyped viral entry into ACE2-HEK293T cells treated with 10 μM of cefaclor, cefotetan, oxytocin, and risedronate sodium, respectively. Data were presented as mean ±SEM (*n* = 3) (* *p* < 0.05; ** *p* < 0.01; *** *p* < 0.001).

The effects of cefaclor, cefotetan, oxytocin, and risedronate sodium on ACE2 HEK293T cell viability were determined, and results are shown in Figure 6A. The cell viability of ACE2 (angiotensin-converting enzyme 2)-HEK293T cells treated with cefotetan and oxytocin was significantly reduced at higher concentrations. The virus inhibition effect of molecules on the cellular level was assessed by detecting the entry of SARS-CoV-2 spike pseudotyped virus into ACE2-HEK293T cells (Figure 6B). The control group was the luciferase luminescence of ACE2-HEK293T infected only with the SARS-CoV-2 spike pseudotyped virus, and the value was normalized. The capacity of the SARS-CoV-2 spike pseudotyped virus to infect ACE2-HEK293T cells was significantly reduced by four medications, and the entry percentage of control was 80% for cefaclor, 72% for cefotetan, 57% for oxytocin, and 54% for risedronate sodium at 10 μM.

### 3.6. The Binding Model Simulation of Active Compounds with Mpro

An analysis of the binding modes of common drug candidates (oxytocin, risedronate sodium, trifluridine, and sodium salt of valproic acid) and 8 cephalosporins (cefaclor, cefepime, cephalothin, ceftriaxone, cefoselis, cefotetan, cefuroxime, and cefotaxime) complexed with Mpro and PLpro revealed favorable interactions between the ligands and target proteins.

**Figure 7:**
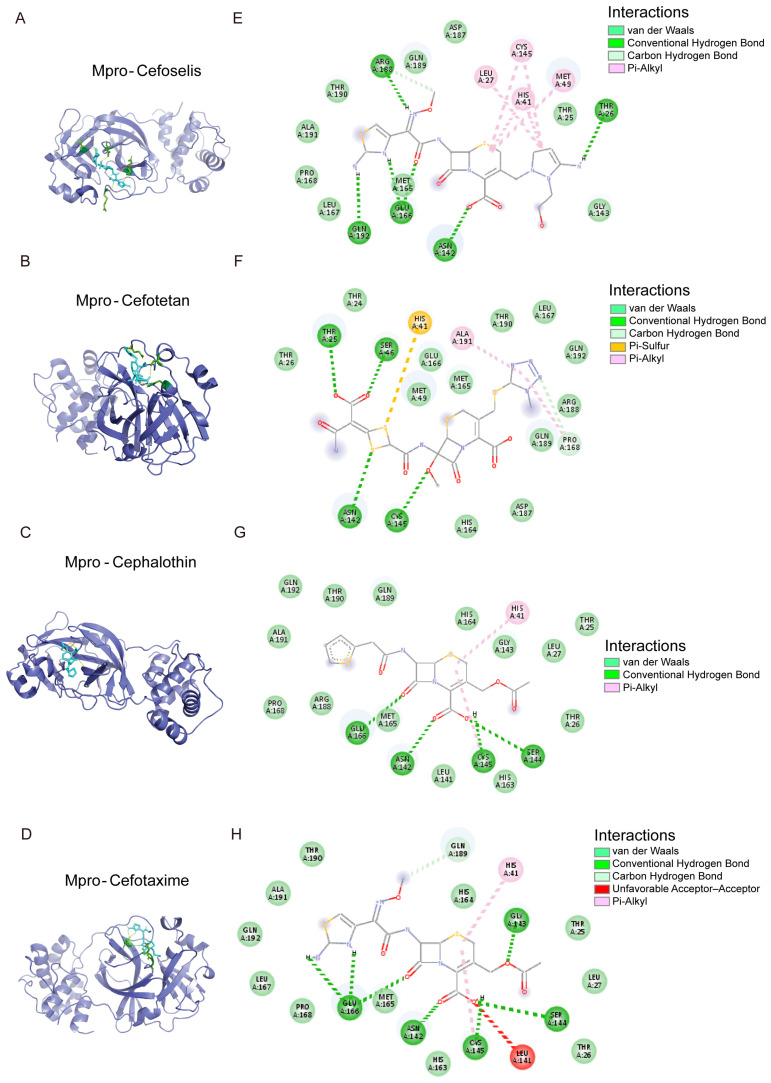
The molecular docking mode and docking site visualization of 4 cephalosporin drugs with Mpro that occupy the catalytic residues CYS145 and HIS41. (**A**–**H**) The molecular docking mode and the docking site visualization of Mpro with cefoselis, cefoteten, ceftriaxone, and cefotaxime. The green dashed lines represent van der Waals; the dark green dashed lines represent Conventional Hydrogen Bond; the light green dashed lines represent Carbon Hydrogen Bond; the pink dashed lines represent Pi-Alkyl; the orange dashed lines represent Pi-Sulfur; the red dashed lines represent unfavorable Acceptor–Acceptor.

**Figure 8:**
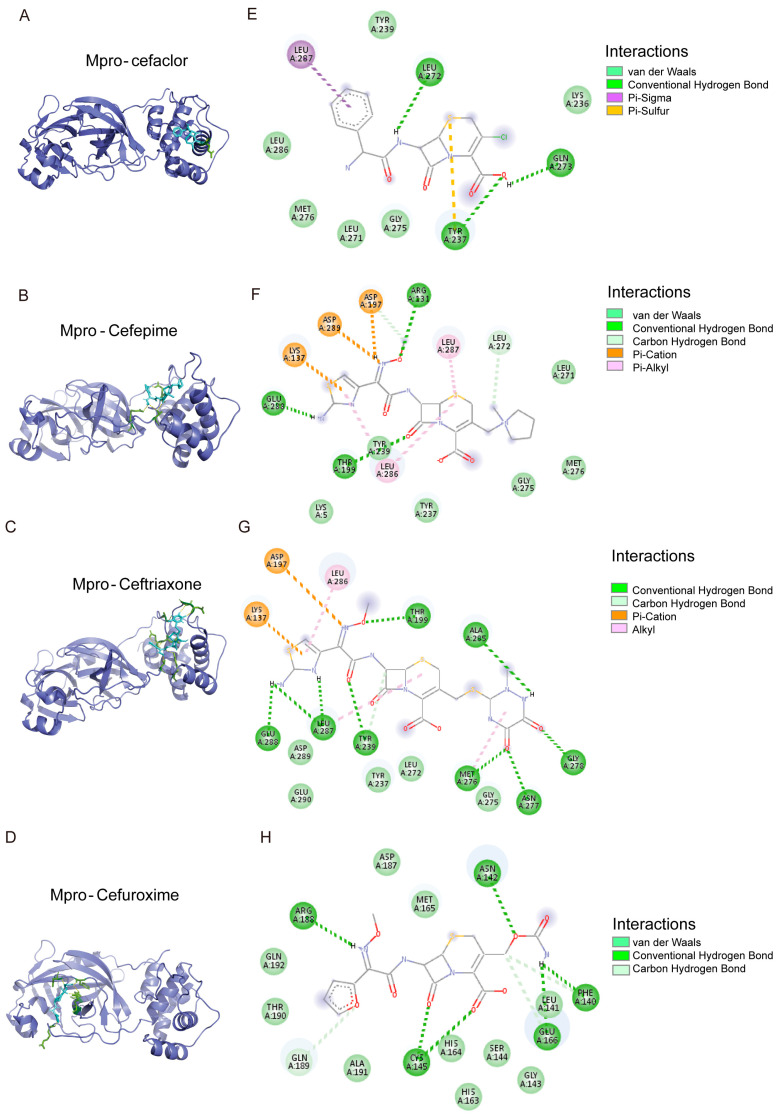
The molecular docking mode and docking site visualization of 4 cephalosporin drugs with Mpro that do not or half occupy the catalytic residues CYS145 and HIS41. (**A**–**H**) The molecular docking mode and the docking site visualization of Mpro with cefaclor, cefepime, cephalothin, and cefuroxime.The green dashed lines represent van der Waals; the dark green dashed lines represent Conventional Hydrogen Bond; the light green dashed lines represent Carbon Hydrogen Bond; the purple dashed lines represent Pi-Sigma; the orange dashed lines represent Pi-Sulfur; the dark orange dashed lines represent Pi-Cation; the pink dashed lines represent Alkyl or Pi-Alkyl.

As depicted in Figure 7, CYS145 and HIS41, the catalytic residues of Mpro, were in proximity to cefoselis, cefotetan, cephalothin, and cefotaxime. Cefuroxime showed interaction with CYS145 only, whereas cefaclor, cefepime, and ceftriaxone did not show any strong interaction (Figure 8). Cefoselis and ceftriaxone also bonded to the active sites, respectively, which are THR26 and SER144.

**Figure 9:**
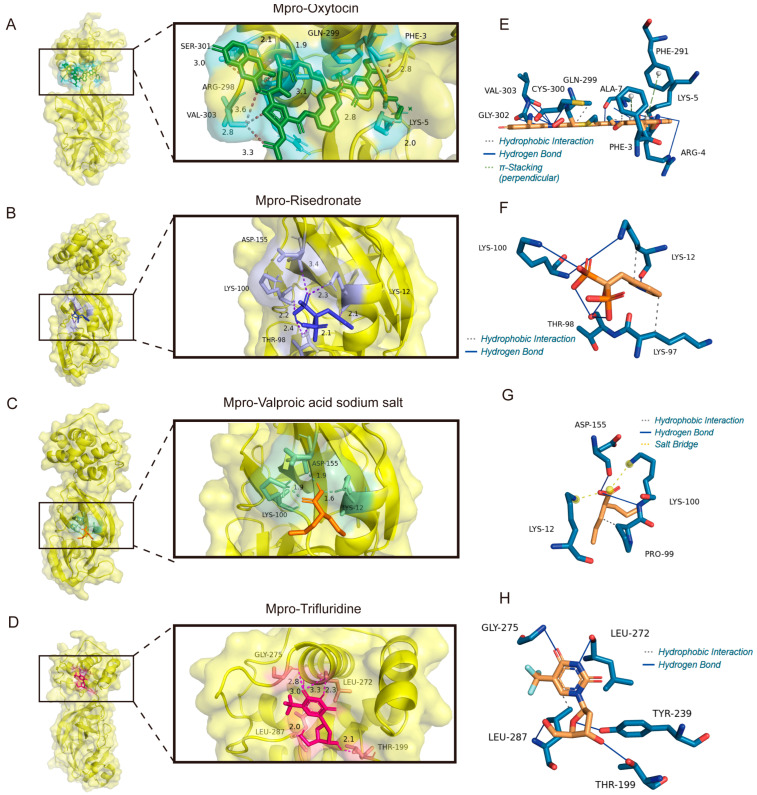
The molecular docking mode and docking site visualization of 4 potential ligands with Mpro. (**A**–**D**) The molecular docking mode of Mpro with oxytocin, risedronate sodium, trifluridine and the sodium salt of valproic acid. (**E**–**H**) The docking site visualization of Mpro with oxytocin, risedronate sodium, trifluridine and the sodium salt of valproic acid. The grey dashed lines represent Hydrophobic Interaction; the blue lines represent Hydrogen Bond; the blue dashed lines represent π–Stacking; the yellow dashed lines represent Salt Bridge.

In Figure 9A,E, the peptide hormone oxytocin may bind to Mpro through perpendicular π stacking with the benzene rings on PHE3 and PHE219, hydrophobic interactions with residues GLN299 and ALA7, and hydrogen bonds might link to CYS300, SER301, GLY302, VAL303, LYS5, and ARG4. Risedronate, a bisphosphonate drug for osteoporosis, may stabilize the binding site through hydrogen bonds with LYS100, ASP155, THR98, and LYS12 and hydrophobic interactions with LYS12 and LYS97 (Figure 9B,F). The hydrogen bonds between ASP155 and LYS100, hydrophobic interactions with LYS12, and salt bridges built through the charge centers of LYS100 and LYS12 constituted the interactions between the antiepileptic drug Sodium salt of valproic acid and Mpro (Figure 9C,G). Trifluridine not only bound firmly to residues GLY275, LEU272, LEU287, THR199, and TYR239 through hydrogen bonds but also bound to residue LEU287 through carbon–hydrogen bonds (Figure 9D,H), which is similar to the binding sites of cefaclor and cefepime.

### 3.7. The Binding Model Simulation of Active Compounds with PLpro

**Figure 10:**
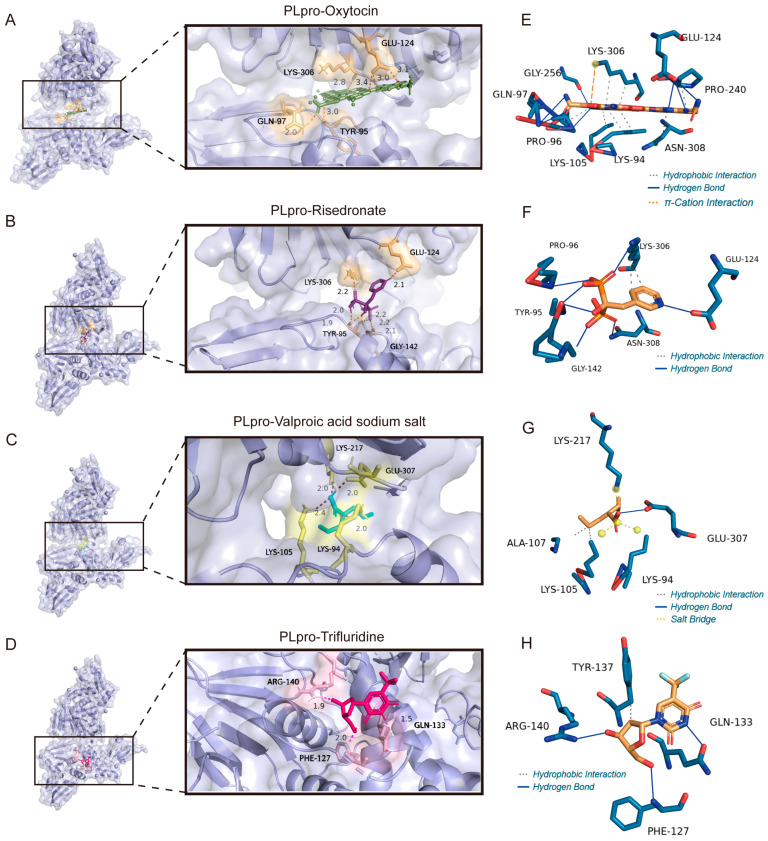
The molecular docking mode and docking site visualization of 4 potential ligands with PLpro. (**A**–**D**) The molecular docking mode of PLpro with oxytocin, risedronate sodium, trifluridine, and the sodium salt of valproic acid. (**E**–**H**) The docking site visualization of PLpro with oxytocin, risedronate sodium, trifluridine, and the sodium salt of valproic acid. The grey dashed lines represent Hydrophobic Interaction; the orange dashed lines represent π–Cation Interaction; the blue lines represent Hydrogen Bond; the yellow dashed lines represent Salt Bridge.

After determining the interactions between microarray active molecules with Mpro, the molecular docking between drug candidates with PLpro was also conducted. As depicted in Figure 10A,E, oxytocin may stabilize the PLpro of SARS-CoV-2 by π-cation interaction with the canonical vesicle of LYS306, hydrophobic interactions with residues LYS306, PRO96, LYS105, LYS94, ASN308 and PRO240, and hydrogen bonds linked to GLN97, PRO96, GLY256 and GLU124. Risedronate occupied the binding site of PLpro through hydrogen bonds with LYS306, PRO96, TYR95, GLY142, ASN308, and GLU124, along with hydrophobic interactions with LYS306 (Figure 10B,F). Sodium salt of valproic acid was bound to PLpro via hydrogen bonds with GLU307, hydrophobic interactions with ALA107 and LYS105, and salt bridges built through the charge centers of LYS217 and LYS94 (Figure 10C,G). Furthermore, hydrogen bonds between GLN133, PHE127, and ARG140 may help stabilize trifluridine in the pocket.

## 4. Discussion

Due to the emergence of variants and drug-tolerant mutants, the SARS-CoV-2 virus is more easily transmitted, and symptoms are more difficult to control. Therefore, COVID-19 is still regarded as a threat to the world’s population. Next-generation therapy designs based on targets other than non-conserved regions, such as the spike protein [25,26], are fundamental for developing novel antiviral drugs. Except spike protein, cysteine hydrolases PLpro and Mpro [27] are also important targets. Mpro and PLpro produce 16 NSPs that play critical role in viral replication and transcription at the host cell membrane, leading to viral multiplication and inflammatory symptoms [28].

Thus, finding Mpro and PLpro inhibitors is a potential way to build antivirus drugs. Small-molecule microarray is a cutting-edge technology that enables the high-throughput screening of thousands of compounds on a small chip [15,17]. Enzymatic assays verified the inhibitory effects of the small molecules, and SARS-CoV-2 spike pseudotyped virus assay determined the inhibitory effect of molecules on virus invasion. Molecular docking is frequently applied for in silico analysis of the binding affinity between ligands and proteins [22]. These technologies provide a new perspective on potential inhibitors that interact with Mpro and PLpro.

Microarray and molecular docking results showed that cephalosporins possess excellent function in Mpro inhibition, and peptide drug oxytocin and risedronate sodium showed an inhibitory effect on both Mpro and PLpro. Although oxytocin did not exhibit the best inhibitory effect, it has been clinically used for various treatments. For instance, oxytocin was used to analyze the effects and neuroendocrine mechanisms of psychophysical therapies for combating post-COVID-19 syndrome [29]. It has been reported that endocrine disorders are important symptoms of patients with COVID-19. Hormones, including oxytocin, can stabilize immune-metabolic processes, inhibit severe inflammatory disorders, and reduce pathogen infection [30,31,32]. As previously reported, oxytocin is secreted from our bodies in several ways, and it can be utilized before the pandemic as a prevention [33,34] or afterward as a long-term treatment for long-COVID [30,35]. It can also be used as a psychological therapy for pandemic-associated stress, increased inflammation levels, immune system imbalance, and cardiovascular disorders [36,37,38]. Oxytocin’s molecular mass is greater than one kDa, and the optimal dosage and mode of administration can be determined by designing a clinical trial. Understanding the interaction between oxytocin and immune factors could help with future medication development. While risedronate sodium was reported as an immunological enhancement, which might protect from SARS-CoV-2 challenge [39]. As previously reported, the modified risedronate might boost vaccine adjuvant effect and long-term humoral immunity [40], and amino-bisphosphonates (N-BPs), including risedronate, have been proven to decrease inflammation, cancer incidence, and respiratory infection [41].

An enzymatic evaluation of small-molecule inhibitors targeting the SARS-CoV-2 main protease (Mpro) and papain-like protease (PLpro) was conducted. Our in vitro enzymatic assays reveal several unexpected and pharmacologically relevant inhibitory activities among clinically approved drugs, offering promising avenues for drug repurposing.

First of all, cephalosporins were regarded as potent Mpro inhibitors [42]. The most striking finding is the robust inhibition of Mpro by multiple cephalosporin antibiotics. Cefotetan and cefaclor demonstrated high inhibitory rates (85% and 79.5%, respectively) at low millimolar concentrations (1–2.5 mM), with IC_50_ values in the low micromolar range (31.10 μM and 71.84 μM). Though further structural studies are needed to confirm binding mechanisms, given their established safety profiles, cephalosporins like cefotetan warrant immediate evaluation in in vivo models of SARS-CoV-2 infection.

Secondly, non-antibiotic drugs are potential drug candidates for dual-target inhibition. Oxytocin and risedronate sodium showed moderate but consistent inhibition of both Mpro and PLpro: With an inhibition rate of 50.4% at 2.5 mM for Mpro and 53.93% at 10 μM for PLpro, oxytocin inhibited both proteins. Regarding risedronate sodium, the inhibition rates for Mpro and PLpro were, respectively, 71.5% at 1.25 mM and 27.85% at 10 μM. Nevertheless, their dual-inhibitory activity could provide synergistic antiviral benefits.

Fortunately, oxytocin, risedronate sodium, cefaclor, and cefotetan each exhibited notable inhibitory effects on pseudotyped virus entry. The pseudotyped virus assay is widely considered to closely mimic SARS-CoV-2 infection and serves as a reliable model for high-throughput drug screening. This is supported by previous studies showing that cephalosporin antibiotics can inhibit the interaction between SARS-CoV-2 and the ACE2 receptor [43], and that oxytocin may also block viral invasion [37]. Unexpectedly, our results demonstrated that risedronate also significantly reduced pseudotyped virus entry.

All cephalosporin drugs, including cefaclor, cefepime, cephalothin, ceftriaxone, cefoselis, cefotetan, cefuroxime, and cefotaxime, showed affinity to Mpro. Surprisingly, Cefoselis, cefoteten, cephalothin, and cefotaxime bind to Cys145 and His41 through hydrogen bonds or alkyl hydrophobic interactions. Cys145 and His41 are believed to have generated the Mpro active location, whereas the cleavage site after LXGG in viral proteins can be cleaved by PLpro, which has a domain made up of Cys111, His272, and Asp286 for the catalyzation [44]. Cephalosporin drugs showed strong interactions and inhibitory effects on SARS-CoV-2 in previous in silico and in vitro studies [45,46]. Nevertheless, even clinically licensed medications that were expected to inhibit Mpro, such as hydroxy-chloroquine, chloroquine, and remdesivir, did not show competitive docking scores [47], which was in agreement with previous reports. The binding affinity of ligands with PLpro ranged from −10.47 to −2.77 kcal/Mol. Tyrphostin 9 decreased mite growth, minoxidil functions as an androgen inhibitor, and diminazene has anti-trypansome properties, among the chemical compounds attached to PLpro. In general, although cephalosporin may interact with Mpro, no specific binding to PLpro was observed.

However, limitations exist. The potent Mpro inhibition by cephalosporins expands the repertoire of FDA-approved SARS-CoV-2 protease inhibitors beyond antivirals. Cefotetan—a widely available antibiotic with favorable pharmacokinetics—emerges as a high-priority candidate for in vivo validation, but cephalosporins indeed struggle to enter the cytoplasm to interact with Mpro [48]. Oxytocin undergoes rapid degradation by oxytocinase following systemic administration, and its classic pharmacological effects are mediated through binding to the OXTR receptor on cell surfaces [49]. Thus, oxytocin’s susceptibility to degradation and the poor transmembrane capacity of cephalosporins represent significant challenges for their systemic administration.

For future work, theoretically and technically, delivering oxytocin or cephalosporins via encapsulation in exosomes or liposomes stands as one of the most promising strategies to overcome these inherent limitations and facilitate cellular entry [50]. The proposed compound may exert potential antiviral effects through encapsulation in liposomes or exosomes, which do not heavily rely on free cytoplasmic diffusion. The pharmacological effects of the encapsulated molecules also require further investigation.

## 5. Conclusions

In summary, this study provided two reliable high-throughput screening systems and binding affinity information to identify small molecules that interact with Mpro and PLpro. Oxytocin and risedronate sodium showed an inhibitory effect on both Mpro and PLpro, and cephalosporins were drug candidates as Mpro inhibitors. Oxytocin, risedronate sodium, cefaclor and cefotetan showed inhibitory effects on SARS-CoV-2 spike pseudotyped virus invasion. The study can help reduce the clinical trial time during a pandemic outbreak, which can be used to expand the usable drug library to combat viral variants and drug resistance, thereby helping prepare for a recurrence of the pandemic.

## Data Availability

Data will be made available on request.

## Supplementary Materials

The following supporting information can be downloaded at: https://www.mdpi.com/article/10.3390/v17121564/s1, Figure S1: The quality control of proteases for microarray embedding; Figure S2: Cytotoxicity of drugs for repurposing; Table S1: Microarray-positive candidates for Mpro and PLpro; Table S2: Protein PDBs for docking.

## Abbreviations

The following abbreviations are used in this manuscript:

Mpro	Main protease
PLpro	Papain-Like protease
GSDMD	Gasdermin-D
ISG15	Ubiquitin-like protein ISG15
NSP5	Nonstructural protein 5
NSP3	Nonstructural protein 3
