# Supplementary material for: Targeting SARS-CoV-2 Mpro and PLpro by Repurposing Clinically Approved Drugs
Source: Viruses. 2025 Nov 29;17(12):1564. doi: 10.3390/v17121564 (PMC12737678; doi:10.3390/v17121564)
Supplement: Supplementary file 1 [file viruses-17-01564-s001.zip › Table S2. protein PDBs for docking.pdf]

Table S2

Protein	PDB	Description	Deposit
main protease	8DZB	Crystal structure of the SARS-CoV-2 (COVID-19) main protease in complex with inhibitor 11	2022-08-06
Papain-Like Protease	7TZJ	SARS CoV-2 PLpro in complex with inhibitor 3k	2022-02-15
