# Supplementary figures and images for: Targeting SARS-CoV-2 Mpro and PLpro by Repurposing Clinically Approved Drugs
Source: Viruses. 2025 Nov 29;17(12):1564. doi: 10.3390/v17121564 (PMC12737678; doi:10.3390/v17121564)

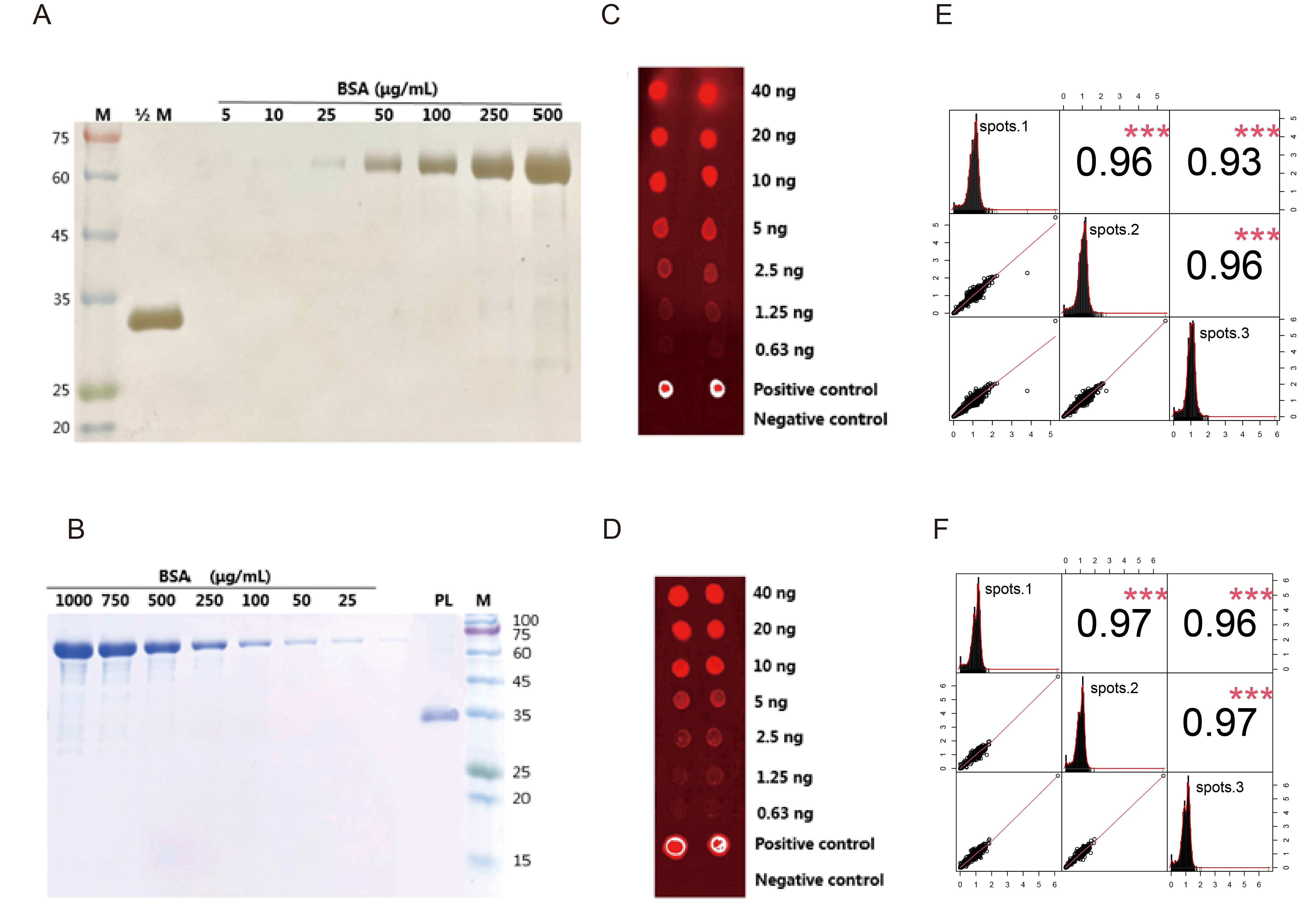

Supplement: Supplementary file 1 [file viruses-17-01564-s001.zip › Figure S1. The quality control of proteases for microarray embedding.jpg]

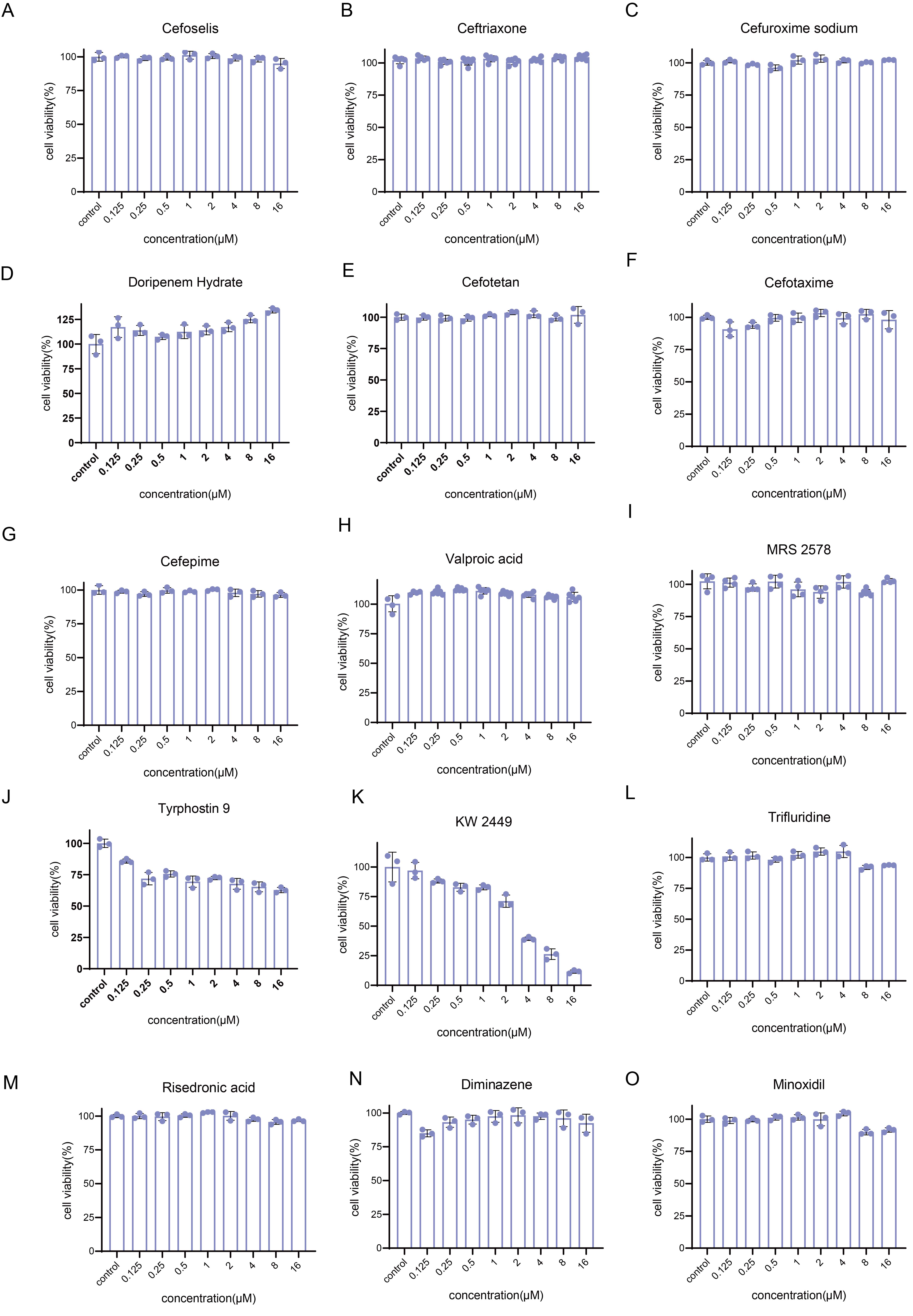

Supplement: Supplementary file 1 [file viruses-17-01564-s001.zip › Figure S2. Cytotoxicity of drugs for repurposing.jpg]
